# Effect of magnetically simulated zero-gravity and enhanced gravity on the walk of the common fruitfly^†^

**DOI:** 10.1098/rsif.2011.0715

**Published:** 2012-01-04

**Authors:** Richard J. A. Hill, Oliver J. Larkin, Camelia E. Dijkstra, Ana I. Manzano, Emilio de Juan, Michael R. Davey, Paul Anthony, Laurence Eaves, F. Javier Medina, Roberto Marco, Raul Herranz

**Affiliations:** 1 School of Physics and Astronomy, University of Nottingham, Nottingham NG7 2RD, UK; 2 School of Biosciences, University of Nottingham, Loughborough LE12 5RD, UK; 3 Centro de Investigaciones Biológicas (CSIC), C/Ramiro de Maeztu, 9 28040 Madrid, Spain; 4 Departamento Fisiología, Genética y Microbiología, Universidad de Alicante (San Vicente del Raspeig), Apartado de correos 99, 03080 Alicante, Spain; 5 Departamento de Bioquímica, Universidad Autonoma de Madrid (UAM), C/Arzobispo Morcillo, s/n 28029 Madrid, Spain

**Keywords:** diamagnetic levitation, microgravity, *Drosophila melanogaster*, motility, diffusion

## Abstract

Understanding the effects of gravity on biological organisms is vital to the success of future space missions. Previous studies in Earth orbit have shown that the common fruitfly (*Drosophila melanogaster*) walks more quickly and more frequently in microgravity, compared with its motion on Earth. However, flight preparation procedures and forces endured on launch made it difficult to implement on the Earth's surface a control that exposed flies to the same sequence of major physical and environmental changes. To address the uncertainties concerning these behavioural anomalies, we have studied the walking paths of *D. melanogaster* in a pseudo-weightless environment (0g*) in our Earth-based laboratory. We used a strong magnetic field, produced by a superconducting solenoid, to induce a diamagnetic force on the flies that balanced the force of gravity. Simultaneously, two other groups of flies were exposed to a pseudo-hypergravity environment (2g*) and a normal gravity environment (1g*) within the spatially varying field. The flies had a larger mean speed in 0g* than in 1g*, and smaller in 2g*. The mean square distance travelled by the flies grew more rapidly with time in 0g* than in 1g*, and slower in 2g*. We observed no other clear effects of the magnetic field, up to 16.5 T, on the walks of the flies. We compare the effect of diamagnetically simulated weightlessness with that of weightlessness in an orbiting spacecraft, and identify the cause of the anomalous behaviour as the altered effective gravity.

## 1. Introduction

Life has evolved under the gravitational field of Earth since it began; so it is fascinating and fundamental to find out how living things respond to an environment with different gravity. Experiments on *Drosophila melanogaster*, the common fruitfly, in microgravity conditions on-board the Columbia Space Shuttle (STS-65) [1] and during the Cervantes mission on the International Space Station (ISS) [2] showed a striking increase in the frequency of locomotor activity and walking speed, compared with controls performed on the ground.

*Drosophila melanogaster* is an ideal model organism on which to study the effects of gravity: the flies are small enough that many individuals can be contained in compact cells suitable for space-flight, yet complex enough to possess a sophisticated gravity sense mechanism [3]. Their use is ubiquitous in studies of biological developmental processes and in endeavours to understand cellular mechanisms in higher organisms, and they have been used in a number of studies on the origin of the biological gravity sense mechanism [4,5]. The motility of these flies has been linked to molecular ageing responses that could be of significance for future human space exploration [6].

In these experiments, we set out to investigate the walks of *D. melanogaster* (henceforth referred to as ‘fruitflies’) in a ground-based ‘simulation’ of the microgravity environment in space. We have used a relatively new technique called diamagnetic levitation to provide a pseudo-weightless environment, which requires a powerful magnetic field with a large field gradient to levitate the flies. Ground-based experiments are essential for selecting feasible and interesting experiments for space-flight studies. A primary aim of our experiments is to demonstrate the usefulness of diamagnetic levitation as a viable alternative to more established ground-based techniques for simulating the effects of microgravity on a complex organism, such as the random positioning machine, or parabolic flights.

An additional aim of the experiments is to validate findings of the original space-flight experiments on fruitflies. In the experiments aboard the Columbia Space Shuttle [1], six groups of 50 male flies remained in orbit for nearly 15 days. The flies were hatched and incubated on the ground until they reached the adult stage. Approximately 8 h after launch, two of the groups were installed in a 1*g* centrifuge aboard the Shuttle. Every 2 days the containers were transferred to a glove box, where they were recorded with a video camera for 15 min to observe their behaviour. All groups of flies (including those from the 1*g* centrifuge) showed pronounced increases in the frequency of locomotor activity and walking speed. It was necessary to remove the flies from the 1*g* centrifuge aboard the Shuttle during periods of video recording. Hence, the centrifuge cannot be regarded as a control in these experiments, at least as far as the behaviour is concerned. Any behavioural abnormalities were identified by comparison with experiments performed on the ground. Although great efforts were made to ensure a close match between the environmental conditions of the flies on the ground and those of their counterparts on the Shuttle, the flies on the ground did not experience the conditions of the Shuttle launch, which were difficult to reproduce exactly in the ground-based experimental control. Indeed, subsequent experiments performed on the ISS showed that the behaviour of the flies on the ISS was sensitive to launch procedures [2]. There is thus some doubt about the root cause of the observed motility increase in microgravity.

By using diamagnetic levitation, we were able to ensure that all groups of flies were treated in the same way, except for their differing positions in the magnetic field, and that all experiments were performed simultaneously.

In diamagnetic levitation, diamagnetic materials such as water and many organic-based materials including oils, plastics and biological material are levitated using a strong, spatially varying magnetic field [7]. Diamagnetic material is weakly repelled from magnetic fields, compared with the more commonly known ‘magnetic’ (i.e. ferromagnetic) materials such as iron, which are strongly attracted to a magnetic field. The diamagnetic force, balancing the weight of the levitating object, acts at the molecular level throughout the body of the object, just as the centrifugal force balances the gravitational force on an object in Earth orbit. The forces on a diamagnetically levitated object differ importantly from those on a floating, neutrally buoyant object (such as the forces on an SCUBA diver), in that the weight of the levitated object is balanced throughout the body of the object, not just at its surface.

Levitation of water and organic materials was reported first by Beaugnon & Tournier [8,9]. The potential of diamagnetic levitation for studying living organisms in weightlessness was first demonstrated by Valles *et al.* [10,11] who levitated frog embryos, and by Geim and colleagues, who levitated a variety of small living organisms, including a live frog ([12–14], see also [15]). Levitation has since also been used in studies of micro-organisms [16–19], single-cell cultures [20–23], biomolecule aggregation *in vitro* [24] and protein crystal growth [25].

We used a superconducting solenoid magnet with a vertical bore to levitate fruitflies. Water, being diamagnetic, is repelled by the strong magnetic field generated by the solenoid, with a force given by the product of the magnetic field strength and the field gradient [13]. Because the field is strongest in the central region of the solenoid bore, the diamagnetic force acts in the direction opposite to gravity in the upper region of the bore, and in the same direction as gravity in the lower region. In our magnet, when the magnetic field at the geometric centre of the solenoid is 16.5 T, water levitates approximately 80 mm above the centre of the solenoid, where the diamagnetic force is equal in magnitude to the gravitational force on it. The technique of stable diamagnetic levitation has been described in detail elsewhere [8,10,13–15,26]. The flies levitate at approximately the same position as water, owing to their high water content (in the region of 70 per cent by mass), as in any animal, and because the dry mass of the insect has a magnetic susceptibility similar to that of water. Further details about the levitation forces are given in §4 and the electronic supplementary material.

Temporal and spatial variations in the walking patterns of fruitflies have been studied as indicators of brain activity in response to environmental cues [27–30]. Here, we concentrate on the measurement of the velocity of the flies and the mean square distance travelled as a function of time, in different effective gravities in the magnetic field.

**Figure 1.**
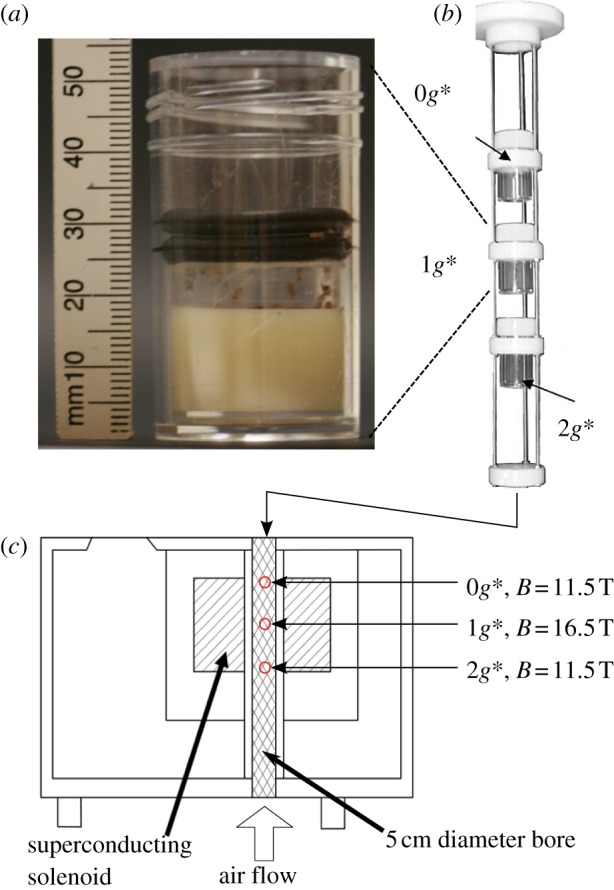
(*a*) One of the arenas contained within a transparent plastic tube (diameter: 25 mm; height: 50 mm), viewed from the side. The arena floor is a semi-solid culture medium (off-white material at the bottom of the tube), which provided a food source and maintained humidity in the arena at close to 100 per cent. The ceiling of the arena is a disc of transparent cellophane (just visible between the two retaining black rubber o-rings in the image, 25–30 mm above the tube's base) punctured around its perimeter to allow gas exchange. (*b*) Three arenas held in a scaffold, before insertion into the magnet bore. (*c*) Each tube was lit around its circumference by six white light-emitting diodes (LEDs), imaged from above by a charge-coupled device (CCD) camera, and its temperature monitored by a thermocouple. The lighting, cameras and temperature sensors have been removed from the scaffold in these images for clarity. (Online version in colour.)

The fruitflies were confined within three cylindrical ‘arenas’, 25 mm in diameter and 10 mm tall, stacked inside the magnet bore, one at the centre of the solenoid, one near the top of the solenoid, and one near the bottom, as shown in figure 1. Flies in the central arena experienced normal gravity. In the arena located near the top of the solenoid, where the diamagnetic force balances the gravitational force, flies experienced pseudo-weightless conditions. Below the normal gravity arena was a pseudo-hypergravity arena where the gravitational and magnetic forces sum together so that the effective weight of the flies is twice that outside the magnet [18]. For convenience, we label the three arenas inside the bore as 0g*, 1g* and 2g*, as shown in figure 1; the asterisk indicates the presence of a strong magnetic field (16.5 T in 1g*, 11.5 T in 0g* and 2g*). We label a zero magnetic field control, outside the magnet, as ‘1*g*’. The flies could not escape from the arenas. We used the calculated effective gravity of water in the magnetic field, computed from the solenoid geometry [26] and measurements of the magnetic field, to determine the vertical position of each of the three arenas in the magnet bore. The 0g* arena was placed to enclose the stable levitation point of water [26]. The 1g* and 2g* arenas were placed to enclose the point where the effective gravity on water is 9.8 ms^−2^ and 19.6 ms^−2^, respectively. Additional details about the apparatus are given in §5; details on the calculation of the effective gravity are given in the electronic supplementary material.

## 2. Behaviour of fruitflies in the magnet

For most of the time, the flies remained in contact with the walls, floor and ceiling of the arena. In common with the findings of previous studies [27–29], the flies alternated between stationary periods (during which they may perform other activities, such as grooming) and active periods in which they moved around the arena. A technical problem owing to the slight degradation of the charge-coupled device (CCD) video images in the strong magnetic field prevented us from identifying any differences in more complex behaviour of the flies, such as grooming or wing beating. Nonetheless, major differences in walking speed could be observed clearly in the three different positions in the magnet bore. It is immediately apparent from the video images that the flies in the 0g* arena walked more quickly and more frequently (electronic supplementary material, movie). Although the flies did perform short flights occasionally in all arenas, flights occurred so infrequently in the confined space of the arena that we could obtain no information on the effect of levitation, or of hypergravity, on flight.

A small number of flies, typically two to four at any one time, levitated freely in the 0g* arena, i.e. not in contact with any surface. These flies levitated within 1–2 mm of the observed levitation point of water, owing to the large water content of the flies. Hence, as a first approximation, we assume for the moment that the effective gravity acting on the organism is the same as that acting on water. We shall discuss the limits to this approximation in §4. The exact position of the levitation point depends on the hydration of the flies. In fact, we observed the levitation point changing during the life cycle of the fruitfly, from egg to larvae to pupae to adult, owing to the difference in water content between each stage of the fruitfly's life cycle. We fine-tuned the solenoid current until the stable levitation point of the adult flies was located in the centre of the 0g* arena.

Freely-levitating flies are drawn towards the stable equilibrium levitation point by the combined gravitational and diamagnetic forces on them, but the flies could escape this ‘trap’ easily by taking flight, as can be seen on the video recording. Some flies held in the ‘trap’ remained motionless for several minutes, while others attempted to walk by climbing over their fellows held in the trap. The camera resolution in this position is too poor (owing to the effect of the magnetic field on the camera) to resolve more detailed behaviour.

## 3. Analysis of the walking paths

**Figure 2.**
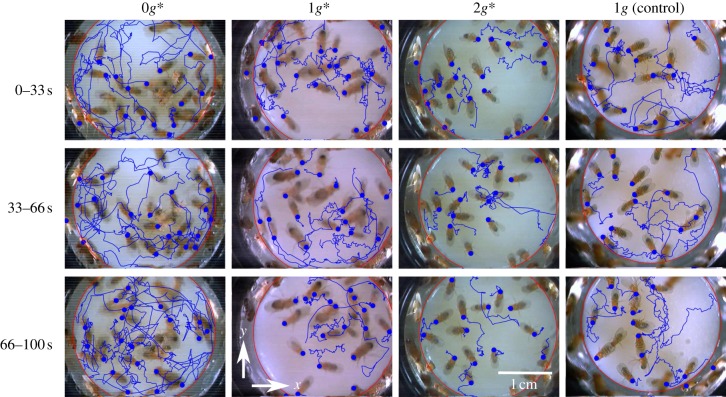
Trajectories of fruitflies walking on the floor of cylindrical arenas (25 mm diameter) shown for three consecutive 33 s periods. The video still-images show the positions of the flies at the start of each period. The position of the head at the start of the period is shown by a dot. The large red circle marks the edge of the arena floor. The flies travelled significantly farther during each period in the pseudo-weightless arena (0g*) than in normal gravity (1*g* and 1g*). The flies travelled shorter distances in pseudo-hypergravity (2g*). The vectors *x* and *y* lie in the horizontal plane, perpendicular to the magnetic field and the direction of gravity (*z*). (Online version in colour.)

Figure 2 shows the walking paths of flies moving over the floor of the arena, observed during three consecutive 33 s periods. Note that these continuous paths include pauses in walking activity when the flies are stationary. During these pauses, the recorded position of the fly does not change, or changes insignificantly, from one video frame to the next. It is immediately apparent that the flies travelled significantly farther, on average, over the arena floor during each 33 s period in the 0g* arena than in normal gravity (1*g* and 1g* arenas), and that the flies travelled shorter distances in 2g*.

### 3.1. Velocity distribution and activity of the flies

From the walking paths, we determined the velocity components of the flies parallel to the arena floor (*v*_*x*_, *v*_*y*_). Here, *v*_*x*_ = *Δx*/*Δt*, where *Δx* is the displacement of the fly along the *x* direction between consecutive video frames and *Δt* = 1/15 s (≈0.067 s) is the time between the capture of one video frame and the next, and similarly for *v*_*y*_. The *x* and *y* directions are labelled in figure 2. Defined this way, the vector (*v*_*x*_, *v*_*y*_) is the mean horizontal velocity of the fly during the interval *Δt*, not the instantaneous velocity of the fly. In determining the velocity, we make no distinction between ‘active’ and stationary periods of the flies, i.e. when the flies are stationary, we simply record the velocity as (*v*_*x*_, *v*_*y*_) = (0, 0). We also determined the speed *u* = (*v*_*x*_^2^ + *v*_*y*_^2^)^1/2^, which is the magnitude of the mean velocity vector of the fly during the time *Δt* between consecutive video frames.

**Figure 3.**
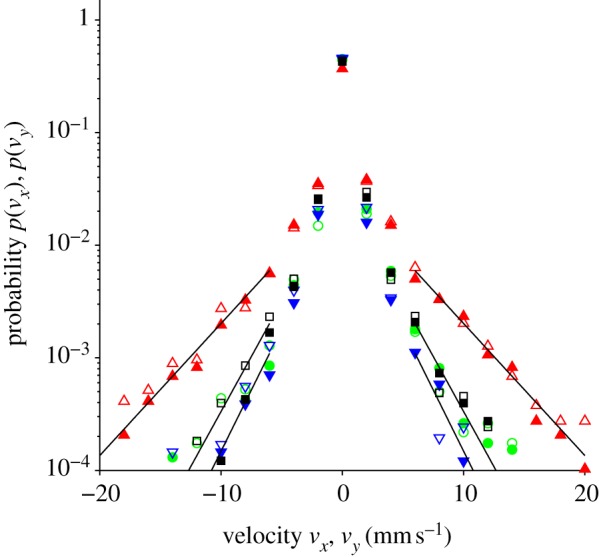
Histogram showing the distribution of the horizontal velocity of fruitflies (*v*_*x*_, *v*_*y*_) in each of the arenas. The distribution of *v*_*x*_ is shown by solid symbols (red triangles, 0g* (11.5 T); green circles, 1g* (16.5 T); blue inverted triangles, 2g* (11.5 T); black squares, 1*g* control (0 T)) and *v*_*y*_ by open symbols. Here *v*_*x*_ = *Δx*/*Δt*, where *Δx* is the displacement of the fly along the *x* direction between consecutive video frames, and *Δt* is the time interval between the capture of one video frame and the next, and similarly for *v*_*y*_. Hence (*v*_*x*_, *v*_*y*_) is the mean velocity of the fly during the interval *Δt*, not the instantaneous velocity of the fly. The data show the distribution for the population, normalized such that *p*(*v*_*x*_)*Δv*_*x*_ is the probability of finding a fly, randomly selected from the population, with a velocity between *v*_*x*_ − *Δv*_*x*_/2 and *v*_*x*_ + *Δv*_*x*_/2 at any particular time; similarly for *v*_*y*_. The bin-width *Δv*_*x*_ of the histograms are 2 mm s^−1^. The solid lines are fits to the tails of the distributions with *p*(*v*_*x*_) ∼ exp(−*b*|*v*_*x*_|); see §4.4. (Online version in colour.)

The histograms in figure 3 show the distributions *p*(*v*_*x*_) and *p*(*v*_*y*_) of the velocity components *v*_*x*_ and *v*_*y*_, respectively, in each arena. The data show the distribution for the population, and are normalized such that *p*(*v*_*x*_)*Δv*_*x*_ is the probability of finding a fly, randomly selected from the population, with a velocity component between *v*_*x*_ − *Δv*_*x*_/2 and *v*_*x*_ + *Δv*_*x*_/2 at any particular time, and similarly for *p*(*v*_*y*_). The histogram bins are *Δv*_*x*_, *Δv*_*y*_ = 2 mm s^−1^ wide. Note that *p*(*v*_*x*_) and *p*(*v*_*y*_), as we have defined them, differ, in general, from the distribution of the *instantaneous* velocity vector (d*x*/d*t*, d*y*/d*t*), and depend on the choice of *Δt*. Nevertheless, they serve as a useful measure of the walking speed of the flies. We observed no significant difference between the distributions *p*(*v*_*x*_) and *p*(*v*_*y*_), showing that there is no bias towards any particular horizontal direction.

**Figure 4.**
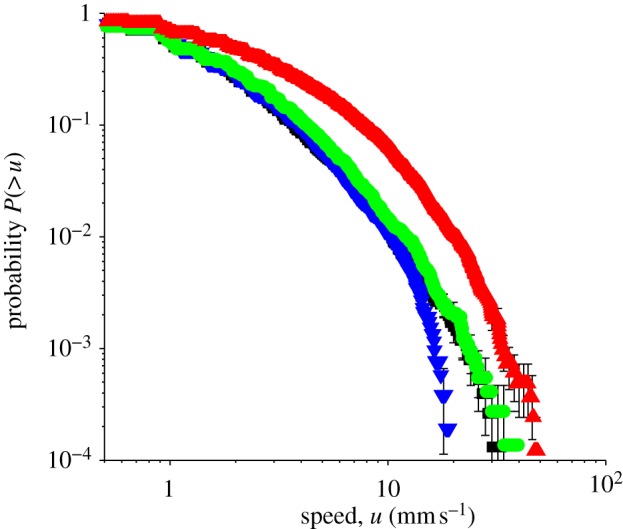
Cumulative histogram showing the distribution of speeds *u* in each of the arenas. The data show the probability of observing a fly (randomly selected from the population) that has a speed greater than *u*. Here *u* = *Δr*/*Δt*, where *Δr* = (*Δx*^2^ + *Δy*^2^)^1/2^ is the distance moved between consecutive video frames, and *Δt* is the time interval between the capture of one video frame and the next. Hence, *u* is the magnitude of the mean velocity of the fly during the interval *Δt*, not the instantaneous speed of the fly. Error bars give an indication of the counting error. Red triangles, 0g* (11.5 T); green circles, 1g* (16.5 T); blue inverted triangles, 2g* (11.5 T); black squares, 1*g* control (0 T). (Online version in colour.)

Figure 4 shows a cumulative histogram plot (sometimes called a rank/frequency plot) of the speed distribution *u*. In this plot, *P*(>*u*) is the probability of observing a fly (randomly selected from the population) that has a speed *greater* than *u*. Unlike the standard histogram, this type of plot does not require us to bin the data, avoiding the need to define a bin width [31].

From the velocity distribution shown in figure 3, we see that the flies in the 0g* arena spent more time walking at higher velocity (on average) than in the other arenas. For example, at any particular moment, it is approximately six to seven times more probable to observe a fly with velocity component |*v*_*x*_| or |*v*_*y*_| between 9 and 11 mm s^−1^ in 0g*, than in 1*g*. It is less probable to observe a fly in 0g* moving with velocity |*v*_*x*_| or |*v*_*y*_| smaller than 1 mm s^−1^, than in the other arenas. When we averaged the velocity (*v*_*x*_, *v*_*y*_) over a larger time *Δt*, we obtained qualitatively similar results: flies in 0g* reached higher velocity more frequently. The difference in velocity distribution between the 1g*, 1*g* and 2g* arenas is less clear from the histogram plot. However, a difference in the walking speeds *u* between flies in 2g* and the normal gravity controls (1g* and 1*g*) becomes apparent in the cumulative histogram shown in figure 4. The probability of observing a fly with speed *u* greater than approximately 10 mm s^−1^ is similar in both 1g* and 2g*, but beyond 10 mm s^−1^, the probability decreases markedly in 2g*, compared with that in 1g* and 1*g*.

Martin studied the temporal frequency of ‘activity’ and ‘inactivity’ in the fruitfly [29]. He considered flies moving greater than 4 mm s^−1^ as ‘active’ and those moving slower than 2 mm s^−1^ as ‘inactive’ (he classed as ‘undefined’, those flies moving between 2 and 4 mm s^−1^). Using this definition of activity, we see from the cumulative histogram that flies in 2g* and 1g* (and 1*g*) are ‘active’ for similar fractions of time, whereas the flies in 0g* spend more than twice as much of their time ‘active’ compared with flies in 1g* and 2g*.

### 3.2. Mean square distance travelled by the flies

**Figure 5.**
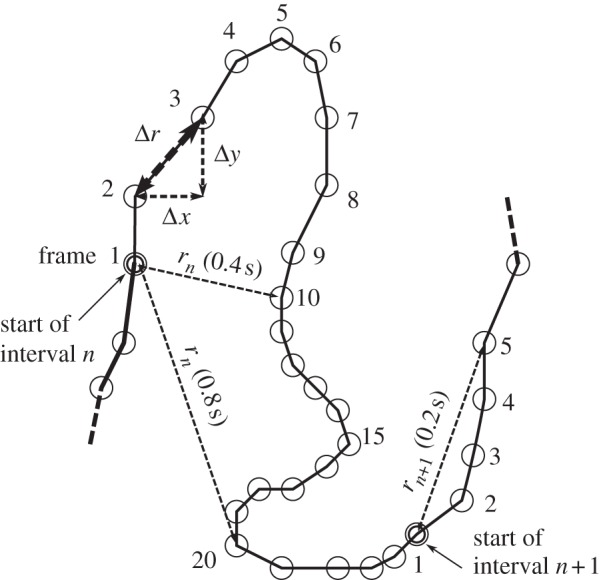
Graphic showing how our measure of the mean square displacement (MSD) of the fruitfly versus time is obtained. Circles represent the position of a single fly in each video frame. Consecutive frames are separated by the time *Δt* = 1/15 s. The period of observation is divided into non-overlapping intervals of 25 video frames each (1.7 s). For each interval *n*, we measure **r**_*n*_(*t*), which is the displacement away from the fly's initial position at the start of the interval (‘frame 1’), as a function of time. The MSD 〈*r*^2^〉 is obtained by averaging the square of the displacement *r*_*n*_^2^ = **r**_*n*_ · **r**_*n*_ over all intervals *n* and all flies. The distance moved between consecutive frames *Δr* is used to obtain the average speed *u* = *Δr*/*Δt*, as described in the text.

**Figure 6.**
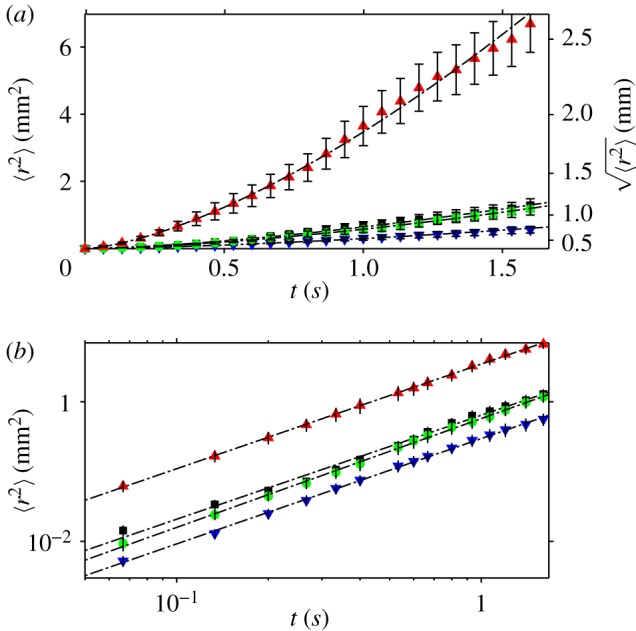
(*a*) MSD 〈*r*^2^〉 as a function of *t* < *τ*, where the period of observation has been divided into *τ* = 1.67 s intervals. The mean is taken over several intervals and all flies. (*b*) The graph of log〈*r*^2^〉 versus log *t* is a straight line, with slope *γ* ≈ 1.5. The lines are offset along the *y*-axis by log*D*. The values of *γ* and *D*, determined from a least-squares fit to the data, are given in table 1. Error bars show the standard deviation of 〈*r*^2^〉. Red triangles, 0g* (11.5 T); green circles, 1g* (16.5 T); blue inverted triangles, 2g* (11.5 T); black squares, 1*g* control (0 T). (Online version in colour.)

**Figure 7.**
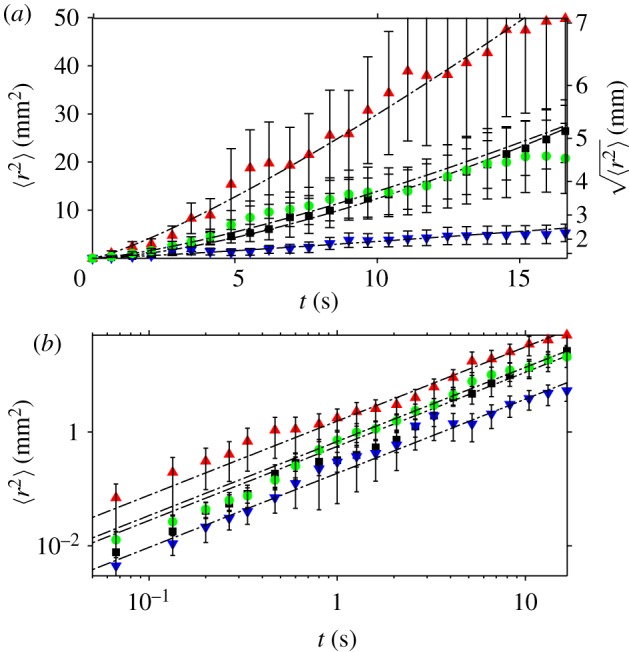
(*a*) MSD 〈*r*^2^〉 as a function of *t* < *τ*, where the period of observation has been divided into *τ* = 16.7 s intervals. The mean is taken over several intervals and all flies. (*b*) Graph of log〈*r*^2^〉 versus log*t*. Error bars show the s.d. of 〈*r*^2^〉. The dashed lines are least squares fit to the data with *γ* = 1.3. Red triangles, 0g* (11.5 T); green circles, 1g* (16.5 T); blue inverted triangles, 2g* (11.5 T); black squares, 1*g* control (0 T). (Online version in colour.)

In the previous section, we determined the mean velocity (*v*_*x*_, *v*_*y*_) of the flies during the time *Δt* between video frames, and the distribution of this velocity in the population. Although one can approach a measure of the distribution of instantaneous velocities of the flies by using a high-speed, high-resolution video camera, this requires intense lighting, which would disturb the behaviour of the flies. An alternative quantitative measure of the walking speed of the flies, which is independent of *Δt*, can be obtained from the mean square displacement (MSD) of the flies as a function of time *t* > *Δt*. We divided the period of observation (100 s) into 20 non-overlapping intervals of 25 frames each, which corresponds to a duration of *τ* = 1.67 s. For each fly and each interval *n*, we measured **r**_*n*_(*t*) · **r**_*n*_(*t*) = *r*_*n*_(*t*)^2^, where the vector **r**_*n*_(*t*) is the displacement of the fly away from its position at the start of the interval, as a function of the time elapsed *t* since the beginning of the interval (figure 5). We calculated the MSD as the average of *r*_*n*_(*t*)^2^ over all intervals *n* and all flies. Within a particular interval, we excluded any trajectories of flies that made contact with the walls. This removed the circular arcs of flies crawling around, or walking on, the walls of the arena, from the analysis. The MSD 〈*r*^2^〉 is shown in figure 6. Error bars show the standard deviation of 〈*r*^2^〉. Note that the maximum variation in the mean square between *individuals* is generally substantially larger than the standard deviation of 〈*r*^2^〉. The graphs show that the MSD of flies in 0g* grows considerably faster with time than flies in 1*g* and 1g*. Conversely, the MSD of flies in 2g* increases more slowly with time than in 1*g* and 1g*. We performed a least-squares fit to the data in figure 6 with the power law 〈*r*^2^〉 = *Dt*^*γ*^, where *D* is an ‘effective diffusivity’. The exponent *γ*, which is the slope of the log–log plot of the data (figure 6*b*), is approximately the same in all gravities, *γ* ≈ 1.5, including outside the magnet. The coefficient *D*, which is directly proportional to the rate at which 〈*r*^2^〉 increases with time, varies considerably with the effective gravity, as is immediately apparent by examining the *y*-intercept of the straight line fits to the log–log plot of the data, as shown in figure 6*b*. The values of *D* obtained from the least-squares fit are given in table 1. Figure 7 shows the MSD calculated using a longer time scale. In these plots, the interval time used in the analysis is *τ* = 16.7 s. Just as in the analysis on the shorter time scale, flies in 0g* increase their MSD more rapidly with time, and more slowly in 2g*, than in 1*g* and 1g*. The MSD of flies in 1g* increases at a similar rate as those in 1*g* in both analyses, as shown in both figures 6 and 7.

**Table 1.** Coefficient *D* and exponent *γ* in the fit to the data 〈*r*^2^〉 = *Dt^*γ*^* shown in figure 6.

	*D* (mm^2^ s^−*γ*^)	*γ*
0g*	3.47 ± 0.03	1.50 ± 0.01
1g*	0.575 ± 0.005	1.56 ± 0.01
2g*	0.297 ± 0.005	1.51 ± 0.01
1*g*	0.64 ± 0.01	1.49 ± 0.02

The analysis that uses intervals of *τ* = 16.7 s generates more noisy data with correspondingly larger error bars, than with *τ* = 1.67 s, as can be seen by comparing figure 7 with figure 6. This is owing to the increased likelihood of any particular fly meeting the arena walls during the longer interval *τ* = 16.7 s: any trajectory that meets the arena walls within time *t* < *τ* is excluded from the measurement of the MSD, thus reducing the size of the set of trajectory data over which the mean is calculated.

## 4. Discussion

The rate at which the MSD increases with time (proportional to *D*) depends on the effective gravity in our experiments: the MSD of the flies grows more rapidly with time in 0g* than in 1g*, and more slowly with time in 2g*. These results corroborate the findings from the experiments performed aboard the Columbia Space Shuttle and the ISS that fruitflies walk more quickly in the microgravity environment and are ‘active’ more frequently. For example, it is twice as likely to observe a fly in 0g* with speed *u* = *Δr*/*Δt* (as defined in §3.1) greater than 4 mm s^−1^ than in the 1g* and 1*g* arenas, at any particular moment.

We also observed that flies in 2g* had a speed distribution similar to those in 1g* and 1*g*, up to approximately 10 mm s^−1^, but beyond this, the probability that flies in 2g* reach higher speed falls relative to the 1*g* and 1g* arenas. For example, the chance of observing a fly in 2g* with *u* greater than 16 mm s^−1^ is approximately three times smaller than in 1g* and 1*g*, at any particular moment. The coefficient *D* of the walks of flies in 2g* is correspondingly smaller than in the other arenas.

In experiments in larger arenas under normal gravity, Valente *et al.* [30] observed a dependence of the velocity distribution on position within the arena, which we do not observe. However, our arena diameter is approximately six times smaller than in Valente *et al.*'s work, in order to fit within the 5 cm diameter bore of the magnet.

### 4.1. Interactions with magnetic field

Before concluding that the anomalous walking speed and activity observed in the magnet can be attributed to the altered effective gravity, we need to consider the differences between the pseudo-weightless condition of diamagnetic levitation and the ‘true’ microgravity environment in orbit—in particular, the effect of the strong static magnetic field (which is, of course, not present in space-flight experiments). In this section, we give a brief overview of magnetic field interactions that can occur in the fruitflies, besides that of the linear diamagnetic force that we use to balance the gravitational force. In §4.2, we discuss the uniformity of the effective gravity acting on the flies.

Mechanical stresses within the organism can be introduced through alignment of biostructures by the magnetic field. Many biological structures contain long sequences of regularly oriented peptide groups. There is a torque on such structures in a magnetic field, owing to the diamagnetic anisotropy of the peptide bond [32]. Some cell structures contain enough of these sequences that the magnetic torque on the structure in relatively weak fields of approximately 1 T can overcome the randomizing effect of thermal motion, resulting in some degree of magnetic alignment [33–36]. Other regularly oriented long chain organic molecules can be aligned by the magnetic field in a similar way [37].

We control for effects of magnetic alignment on behaviour, and any other interactions with the magnetic field, such as magnetohydrodynamics effects [38], by comparing the motion of the flies in the 1g* arena with the flies in the arena positioned well away from the magnet (1*g* arena). The strong 16.5 T magnetic field present in the 1g* arena had no clearly identifiable effect on the flies' walking behaviour: the velocity distribution of flies in 1g* is the same (i.e. within the scatter of the data) as that in 1*g*, and *D* and *γ* characterizing the MSD are similar. Although the exponent *γ* appears to be slightly higher in 1g* from that in the other arenas, the difference in *γ* between 1g* and 1*g* is not large enough, compared with the error in the measurement, to attribute unambiguously to the magnetic field.

Because the fruitfly can sense the Earth's magnetic field [39], it might be expected that the strong field in the superconducting magnet, being of order 10^5^ times larger than the Earth's field at the surface, should disrupt their magnetic sense. Gegear *et al.* [39] reported that flies introduced to a ‘T-maze’ showed different preferences for each arm of the maze depending on their exposure to a magnetic field. Gegear *et al.* used a field of 5 × 10^−4^ T in their experiments, which is 3 × 10^5^ times smaller than that generated by the superconducting magnet we use in these experiments, yet we observe no effect of the field on the walking patterns of the flies. One reason for this discrepancy may be the lighting used in our experiments. Gegear *et al.* showed that the magnetic sense is light-dependent: flies exposed to broad-spectrum illumination displayed sensitivity to the magnetic field, but did not respond when a filter blocking wavelengths of less than 420 nm was employed. In our experiments, the only illumination is by white light emitting diodes (LEDs). Because such lights emit only a small fraction of their energy at less than 420 nm, we speculate that this may explain why we do not observe significant differences in the walking patterns of the flies in the field compared with the control outside the magnet.

According to Faraday's law of induction, electric fields and corresponding electric currents can be generated in the organism if it moves through a static magnetic field with a field gradient, such as is present in the 0g* and 2g* arenas. Glover *et al.* [38] have studied the disorientating effects of such induced currents on human subjects. The induced electric field depends on the rate of change of magnetic flux through the organism, which is proportional to the product of the velocity of the organism and the magnetic field gradient in the direction of motion. Because the direction in which the magnetic field gradient is largest is close to vertical in both the 0g* and 2g* arenas, an electric field can be generated through small vertical motions of the fly as it walks horizontally over the arena floor. Because the magnitude of the magnetic field gradient is the same in the 2g* arena as in the 0g* arena, the magnitude of the induced electric field is the same in both the 2g* and 0g* arenas. Evidently then, the behavioural differences between 0g*, 1g* and 2g* cannot be attributed to an electric field (or associated electric current) induced by linear movement through the magnetic field. An electric field can also be induced by rotational movement (of the head for example) in the magnetic field by the same mechanism [38], but because this can occur even where there is no magnetic field gradient, we can use the comparison between 1g* and 1*g* arenas to conclude that behaviour is not affected significantly by this mechanism.

### 4.2. Effective gravity

We now discuss in more detail the effective gravity acting on the flies. The linear diamagnetic force on the flies is proportional to the product of three quantities: the *magnitude* of the magnetic field (N.B., although the magnetic field is a vector field, its magnitude is a scalar quantity), the spatial gradient of the magnetic field magnitude and the magnetic susceptibility of the material. The magnetic force acts on each molecule of the material. The effective gravitational force is defined as the sum of the linear diamagnetic and gravitational forces, per unit mass; the mathematical definition can be found in the electronic supplementary material.

Unlike the other magnetic field interactions discussed earlier, the effective gravitational force depends on the direction of the magnetic field gradient relative to the direction of the gravitational force. Note that the direction of the magnetic field gradient with respect to gravity is the key difference between the environment in the 0g* arena and the 2g* arena: in the 0g* arena the magnetic field increases in the same direction as the gravitational force (the diamagnetic force acts opposite to the direction of gravity), whereas in the 2g* arena, the field decreases in the direction of gravity (the diamagnetic force acts in the same direction as gravity).

Because the flies levitated close to the levitation point of water (within 1–2 mm) we have, so far, assumed that the effective gravity acting on the flies is approximately the same as that acting on water. We now discuss a number of caveats to this assumption and their significance in these experiments.

On Earth, the weight of a biological organism is supported by mechanical stresses within it. In orbit, or in deep-space, there are no such gravitationally induced stresses. The aim of levitating the organism is to reduce these gravitational stresses to as near to zero as possible by balancing the force of gravity with the linear diamagnetic force, in order to simulate a weightless environment. The effective gravity acting on the levitating material depends on its magnetic mass susceptibility *χ*_*m*_. In a homogeneous material (for example, a well-mixed diamagnetic aqueous solution or organic oil) *χ*_*m*_ is a constant, and hence the effective gravity acting on the material, and the resulting stresses, are relatively simple to calculate [26]. The situation is different for a biological organism. Differences in *χ*_*m*_ between the biological structures within the organism causes the effective gravity to vary from point to point within the organism, generating additional internal stresses. Further details are given in the electronic supplementary material. Fortuitously for our experiments, in the case of soft biological tissues, *χ*_*m*_ differs by only up to approximately 10 per cent from the susceptibility of water [40]. Hence, in many organisms, we expect any influence of the spatial variation in effective gravity to be small compared with the overall reduction in gravity. Valles *et al.* [10] estimated the internal stresses induced in a levitated frogs egg from the measured susceptibilities of a few major cellular constituents. They concluded that the stresses induced by the spatial variation in effective gravity were considerably smaller than (typically approx. 10% of) the gravitationally induced stresses in 1*g*.

In our experiments, we can observe the relative importance of the spatial variation in *χ*_*m*_ by comparing behaviour in the 0g* arena with that in 2g*. The spatial variation in effective gravity, owing to variation in *χ*_*m*_ between tissues, is similar in both the 0g* and 2g* arenas, as explained in the electronic supplementary material. Hence, if the spatial variation of the effective gravity had a significant influence on the flies' behaviour, we would expect to find behaviour in 2g* similar to that in 0g*, or behaviour dependent on the flies' orientation with respect to the direction of the field gradient. However, we observe that walking speed is significantly enhanced in 0g* compared with 2g*, whatever the orientation of the flies in the magnetic field, as can be clearly seen in the electronic supplementary material video. Hence, we conclude that the difference in behaviour between arenas is not significantly influenced by the variation in effective gravity compared with the overall reduction in the effective gravity. This means that, as far as the walks of the flies is concerned, we expect the effects of levitation to be comparable to the effects of space flight or free fall.

Even for a homogeneous substance, like water, there is some spatial variation in the effective gravity, owing to the spatial variation of the magnetic field. By confining the flies inside the arenas, we have restricted the range of the effective gravity acting on water to less than 6 per cent of *g*; the variation within each of the arenas is shown in the electronic supplementary material. The stresses resulting from the spatial variation of the magnetic field can be thought of as tidal forces (the spatial variation, i.e. gradient, of the Moon's gravitational force over the Earth's surface is responsible for the sea tides). In our experiments, the tidal forces are weak compared with the surface tension of water: the effect on the surface oscillations of a levitated cm-scale water droplet are slight [26], suggesting that such tidal forces should have little effect on similarly sized, or smaller, biological organisms.

### 4.3. Response to reduced gravity

We speculate that the enhanced walking speed and activity in the 0g* arena can be explained by a simple physical effect: the flies moved more rapidly and more frequently in 0g* because locomotion expends less energy, there being no work to do against the gravitational force. Consistent with this hypothesis, flies were measurably slower in the 2g* arena than in the 1g* or 1*g* arenas. Detailed studies of the energy efficiency of different modes of insect flight have been performed recently [41,42]. For *walking*, it is clear that the flexing of the flies' joints and muscles would expend less energy in simulated microgravity than 1*g* or 2*g*. Simulation of increased gravity in a centrifuge results in a corresponding decrease in the motility of fruitflies [43].

Alternatively, the abnormal walks observed in the 0g* arena could emerge from a change in the flies' perception of gravity. The flies can respond to gravity using senses that measure the stresses on, and the angle of, joints in the body [3]. Such senses are responsible for directing normal gravitactic behaviour [5]. These senses should perceive the reduction in the weight of the organism. It is also possible that these senses could be influenced by a spatial gradient in effective gravity owing to the differences in magnetic susceptibility between tissues, as discussed in §4.2. Studies investigating this mechanism in human subjects in magnets used for high-field (*B* = 3–7 T) magnetic resonance imaging have shown that the human vestibular system responds to a (static) magnetic field × field-gradient product as low as 1 T^2^ m^−1^ [38] (cf. the 1360 T^2^ m^−1^ required to levitate the flies in our experiments). Nevertheless, even if the gravity sense of flies is affected by stresses resulting from the variation in magnetic susceptibility, these experiments show that the walking speed of the flies, however governed, is primarily influenced by the mean effective gravity, not the spatial gradient in effective gravity, as we have discussed already.

One way to distinguish whether the anomaly in walking speed in 0g* is primarily owing to the flies' altered perception of gravity, or whether it is because walking simply expends less energy in 0g*, would be to compare the walks of different sized flies in different effective gravities. If energy is important, we might expect the anomaly in speed to be more pronounced in more massive flies. Owing to the small number of flies that can be contained simultaneously within the small arenas within the magnet, we were not able to observe statistically significant differences in the motility of different-sized individuals. This would be a good topic for future study, using a centrifuge, for example. Further studies, using altered-gravitropic strains [44], are planned in order to determine the mechanism by which the flies' walk is altered by changes in gravity, or whether several mechanisms are important here.

The observations in this paper were made for flies confined to 25 mm diameter arenas. The size of the arena used in diamagnetic levitation experiments is limited by the size of the magnet bore (and ultimately by the present limit of high magnetic field technology). The earlier experiments on board the ISS and Shuttle demonstrated that the increased walking speed could be observed in larger arenas [1,2], showing that confinement of the flies to such small volumes is not necessary to induce the anomalous behaviour.

### 4.4. Form of velocity distribution and mean square displacement

The velocity distribution of the population, shown in figure 3, is evidently non-Gaussian (i.e. it does not behave like Brownian motion, which has a velocity distribution of the form *p*(*v*_*x*_) ∼ exp(−*av*_*x*_^2^)). The tails of these distributions decay more slowly, as *p*(*v*_*x*_) ∼exp(−*b*|*v*_*x*_|), as shown by the straight line fits to the log plot of the distribution (figure 3). Valente *et al.* [30] have observed that individual flies confined in a circular arena exhibit a non-Gaussian velocity distribution, and the distribution we observe for the population may reflect this. Alternatively, the slower decay can result from pooling the data from a population containing some variation between individuals [45], just as we have done here.

We now discuss briefly our finding that *γ* > 1, which represents a so-called superdiffusive walk. There has been some debate recently on whether or not the movements of a wide variety of animals, including deer, bumblebees and wandering albatross, can be modelled as a special type of random walk, known as a Lévy flight or Lévy walk [46]. Sims [47] show that some marine predators can be modelled this way. Cole found fractal patterns of activity in *D. melanogaster*, which can generate a Lévy flight [27]. Such walks give rise to superdiffusive motion, but this is not the only way that superdiffusion can arise. Temporal correlations in the velocity can also produce superdiffusion [48], as follows. There is a tendency of the flies to maintain their direction of travel over short time scales. At very short time scales, the path of the fly approaches an uninterrupted straight-line (so-called ballistic) motion, for which the MSD increases quadratically with time, i.e. *γ* = 2. With increasing time, the direction of motion becomes less correlated, and *γ* falls accordingly. For a Gaussian random walk, such as Brownian motion, in which there is no correlation between time steps, we have *γ* = 1. Our finding that *γ* falls between 1 and 2 is consistent with the fact that we measure the MSD on a time scale in which we expect the direction of motion to exhibit some correlation in time.

The value of *γ* for the population depends also on the spread in the behaviour of individuals. For example, a population consisting of flies that maintain their direction of travel over relatively long times (i.e. having a relatively long velocity autocorrelation time) and flies that alter their direction frequently (i.e. having a relatively small velocity autocorrelation time) would also generate a *γ* for the population intermediate between 1 and 2.

The movements of the flies are obviously also influenced by their confinement inside the arena (see also [30]): as we increase *τ* up to 17 s, we observe that *γ* falls to approximately 1.3 (figure 7). We might expect *γ* to fall with increasing time scales owing to confinement of the flies by the arena walls, which provides an upper limit to the MSD. From this data, we cannot rule out the possibility that *γ* might fall to 1, when measured over a long enough time scale.

The focus of this paper is to determine the effect of different effective gravities on the walks of the flies, requiring us to use relatively small arenas and relatively small populations in order to fit within the confines of the magnet bore. Experiments performed in large or open arenas with much larger populations would be valuable in determining which of the earlier mentioned possibilities is responsible for the apparent superdiffusive behaviour.

It is striking that the effect that levitation has on the speed distribution is much more pronounced than the effect that pseudo-2*g* hypergravity has, below 10 mm s^−1^. Only above approximately 10 mm s^−1^ does doubling the effective weight of the organism (i.e. in the 2g* arena) begin to limit significantly the frequency that flies exceed a particular speed, compared with flies in normal gravity (1*g*, 1g*). The reason for this is not well understood, because it is a difficult problem to model the relationship between speed and weight. Future experiments are planned at intermediate effective gravities to study this effect.

A corresponding effect is observed in the measurements of MSD. On short time scales, *τ* = 1.67 s, the difference between *D* in the 0g* arena and *D* in the 1g* (and 1*g*) arena is much larger than the difference between *D* in the 2g* arena and 1g* (and 1*g*). When measured over the longer time interval *τ* = 16.7 s, however, the increase in *D* in 0g* is less pronounced: *D* = 1.55, 0.68, 0.19 and 0.58 for the 0g*, 1g*, 2g* and 1*g* arenas, respectively, for *γ* = 1.3. This is an effect of confinement of the flies. In our analysis, a trajectory that meets the arena walls within time *t* < *τ* is excluded from the calculation of the MSD. Doing this removes predominantly those trajectories with larger MSD from the analysis, reducing the corresponding value of *D*. Because the likelihood of any particular trajectory meeting the arena walls is greater for longer intervals *τ* and trajectories with larger MSD, the measured *D* is smaller at longer *τ*, with the greatest reduction of *D* observed in populations with larger MSD.

### 4.5. Comparison with experiments performed in Earth orbit

Our observations agree with experiments performed on the Columbia Space Shuttle and the subsequent mission to the ISS: we observed a pronounced increase in the frequency of locomotor activity and the walking speed of the fruitfly in pseudo-weightless conditions. All the experiments in the magnet and the 1*g* control outside the magnet were performed simultaneously, under the same conditions of atmospheric pressure, temperature, humidity, lighting and with the same batch of fruitflies. As we have discussed earlier, the differences in the observed behaviour within the magnet are owing to differences in the effective weight of the flies. There were no additional effects of the strong magnetic field on the walks of the flies. While there remains the possibility that the magnetic field affects the flies in some other way not revealed by the analysis of walking patterns, our analysis strongly suggests that the weightlessness of microgravity was responsible for the increased motility observed in the original experiments on board the Shuttle and ISS. Although the experiments on the ISS showed some sensitivity to launch procedures, our study indicates that launch procedures are not the cause of the increase in motility *per se*.

### 4.6. Conclusion

This study shows that the walking speed of fruitflies and their ‘activity’ is altered significantly by counteracting the gravitational force. Diamagnetic levitation enabled us to maintain tight control over the experimental conditions of all the experimental subjects. This allowed us to identify, unambiguously, the alteration of effective gravity as the cause of the anomalous behaviour. We have shown how diamagnetic levitation can be used to assess, quantitatively, the behavioural response of a macroscopic organism to zero-gravity.

Four billion years' of evolution have equipped life on Earth to withstand the stresses generated by the ever-present pull of gravity. Here, we have shown that diamagnetic levitation can be used to investigate directly the influence of changing gravity on the locomotion of a complex multi-cellular organism, and that close comparison can be made with experiments performed in space.

## 5. Material and methods

### 5.1. Superconducting magnet

The superconducting solenoid magnet (Oxford Instruments Nanoscience, Abingdon, UK) has a room temperature, vertical, 5 cm diameter bore that is open to the laboratory at both ends. At maximum current, it produces a magnetic field of 16.5 T at the geometric centre of the solenoid. The solenoid is cooled by a closed-cycle refrigeration system, which enables the magnet to run for several months continuously at high fields.

### 5.2. Arenas

The flies were confined to 25 mm diameter, 10 mm tall arenas. The 1g* arena was placed at the centre of the solenoid. The 0g* arena was located 80 mm above the 1g* arena. The 2g* arena was 80 mm below the 1g* arena. Each of the arenas were contained within a transparent plastic specimen tube (diameter: 25 mm; height: 50 mm). The arena floor was formed from a semi-solid culture medium. This is visible as the off-white material at the bottom of the tube in figure 1. The culture medium was poured into the tubes and allowed to set. The culture medium provided a food source for the flies and maintained humidity in the arena at close to 100 per cent. The ceiling of the arena was a disc of transparent cellophane, 10 mm above the floor, retained between two black rubber o-rings. These can be seen in figure 1, 25–30 mm above the base of the tube. The disc was punctured around its perimeter to allow gas exchange. The 0g*, 1g* and 2g* tubes were located in a non-magnetic scaffold and held in place by plastic rings, as shown in figure 1. The scaffold, with the tubes, was inserted into the bore of the magnet. The position of the 0g* retaining ring on the scaffold was adjusted so that the 0g* arena enclosed the levitation point of water. The positions of the 1g* and 2g* rings were similarly adjusted. The experiments inside the magnet were conducted simultaneously with an external control in an incubator (1*g* arena), well away from the magnet.

### 5.3. Fruitflies

Approximately thirty 1–2 day-old Oregon R *D. melanogaster* flies, with equal number of males and females, were sealed into each arena a few hours before the beginning of the observation period, to allow them to acclimatize to the environment inside the magnet.

### 5.4. Lighting

Each tube was lit around its circumference by six equally spaced surface-mount white light (InGa)N LEDs, on a printed circuit board ring attached to the scaffold. The LEDs (OSRAM Optosemiconductors GmbH) had a dispersion angle of 120° and a luminous intensity of 495 mcd, when a 20 mA current was applied. The LEDs provided the only source of light, once the scaffold was located in the magnet bore. Care was taken to avoid ambient light entering the magnet bore.

### 5.5. Temperature control

The temperature of the bore was maintained at 24°C by passing dry air at atmospheric pressure, supplied from a compressor, through the magnet bore at 1 l per second. The temperature of the pumped air was brought up to 24°C, prior to entering the magnet bore, by passing it through a coiled copper pipe immersed in a temperature-controlled water bath. Each plastic specimen tube was fitted with two thermocouples to monitor the temperature. During the experiments, the temperature of the tubes were maintained with less than 0.1°C variation between them. The 1*g* control arena was inserted into a dummy magnet bore: a brass tube with the same dimensions of the bore. The temperature of the dummy bore was maintained at 24°C by placing it in an incubator, placed well away from the magnet.

### 5.6. CCD cameras

Each tube was imaged from above by a CCD camera (Fire-i Digital Board Cameras, Unibrain Inc.), modified for use in high magnetic field, mounted on the scaffold 10 mm above the top of each tube. The cameras recorded video footage of the flies at 15 frames per second (0.067 s per frame). The spatial resolution of the images of the flies is approximately 20 pixels per mm (0.05 mm per pixel) and was calibrated against the dimensions of the tube. The position of the head of each fly was determined for 1500 consecutive frames, i.e. for 100 s of footage, by eye. We recorded only the movement of flies walking on the floor of the arena. We ignored flies walking on the other surfaces, flies not in contact with any surface (i.e. levitating freely), and also flying, which they did infrequently in the confined space of the arena.
